# Old and New Challenges in Uveitis Associated with Behçet’s Disease

**DOI:** 10.3390/jcm10112318

**Published:** 2021-05-26

**Authors:** Julie Gueudry, Mathilde Leclercq, David Saadoun, Bahram Bodaghi

**Affiliations:** 1 Department of Ophthalmology, Hôpital Charles Nicolle, F-76000 Rouen, France; 2 Department of Internal Medicine, Hôpital Charles Nicolle, F-76000 Rouen, France; mat3leclercq@gmail.com; 3 Department of Internal Medicine and Clinical Immunology, AP-HP, Centre National de Références Maladies Autoimmunes et Systémiques Rares et Maladies Autoinflammatoires Rares, Groupe Hospitalier Pitié-Salpêtrière, F-75013 Paris, France; david.saadoun@aphp.fr; 4 Sorbonne Universités, UPMC Univ Paris 06, INSERM, UMR S 959, Immunology-Immunopathology-Immunotherapy (I3), F-75005 Paris, France; 5 Biotherapy (CIC-BTi), Hôpital Pitié-Salpêtrière, AP-HP, F-75651 Paris, France; 6 Department of Ophthalmology, IHU FOReSIGHT, Sorbonne-AP-HP, Groupe Hospitalier Pitié-Salpêtrière, F-75013 Paris, France; bahram.bodaghi@aphp.fr

**Keywords:** uveitis, retinal vasculitis, Behçet’s disease, anti-TNFα agent, tocilizumab, biologics

## Abstract

Behçet’s disease (BD) is a systemic vasculitis disease of unknown origin occurring in young people, which can be venous, arterial or both, classically occlusive. Ocular involvement is particularly frequent and severe; vascular occlusion secondary to retinal vasculitis may lead to rapid and severe loss of vision. Biologics have transformed the management of intraocular inflammation. However, the diagnosis of BD is still a major challenge. In the absence of a reliable biological marker, diagnosis is based on clinical diagnostic criteria and may be delayed after the appearance of the onset sign. However, therapeutic management of BD needs to be introduced early in order to control inflammation, to preserve visual function and to limit irreversible structural damage. The aim of this review is to provide current data on how innovations in clinical evaluation, investigations and treatments were able to improve the prognosis of uveitis associated with BD.

## 1. Introduction

Behçet’s disease (BD) is a systemic inflammatory disease of unknown origin, first described by Hulusi Behçet, a Turkish dermatologist, in 1937. BD is a systemic form of vasculitis of all calibers, involving both arteries and veins, affecting the entire body, at the borderline between autoimmune diseases and auto-inflammatory syndromes [1,2]. BD features include intraocular inflammation, arthritis, oral and genital ulcerations and skin lesions, but multiple visceral localizations (neurological, gastrointestinal and cardiovascular) may also be involved. The evolution is unpredictable, due to more or less regressive flares. Uveitis is one of the most severe complications of the disease [3]. Until the late 1990s, the visual prognosis of patients with BD uveitis was unsatisfactory [4]. Since then, progress in biologic therapy has transformed visual outcomes.

The aim of this review is to provide current data on how earlier diagnoses based on clinical evaluation and investigations as well as therapeutic innovations and strategies have greatly improved the prognosis of uveitis associated with BD.

## 2. Methodology and Literature Search

We conducted an unsystematic narrative review by selecting articles written in English and French from PubMed/MEDLINE database published until March 2021. The keywords used to screen the database were searched in Medical Subject Headings (MeSH) and were: (Behçet’s disease) AND (uveitis) AND (diagnosis) OR (prognosis) OR (therapy), and (Behçet’s disease) AND (biologics) OR (biological agents).

## 3. Epidemiology of Uveitis Associated with Behçet’s Disease

BD has two main epidemiological characteristics that strongly guide the diagnosis. Firstly, it is widespread throughout the world but is particularly present in the Mediterranean basin, the Middle East and Asia, following the Silk Road. Average prevalence rates are 20–420/100,000 inhabitants for Turkey, 2.1–19.5 for other Asian countries, 1.5–15.9 for southern Europe and 0.3–4.9 for northern Europe [5]. Interestingly, immigrants from regions with a high prevalence of BD keep the same high risk close to that observed in their countries of origin, highlighting the important role of genetics in BD [5], yet familial cases are not frequent (less than 5%) [6]. However, a strong link with human leukocyte antigens (HLA), specifically HLA-B51, was found, but the presence of HLA-B51 is insufficient to confirm or invalidate BD diagnosis [7]. The second main epidemiological characteristic is the occurrence of BD in young adults of both genders, most often between 15 and 45 years old. Occasionally, BD can occur in young people below the age of 16 years in 4 to 26% of cases and carries a strong genetic component. Boys have the worst outcomes with more frequent neurological, ocular and vascular disease [8].

## 4. Prognosis of Behçet’s Disease Uveitis over Time

Ocular involvement is common in BD and is potentially severe, as it is sight-threatening. BD uveitis may be responsible for a large number of cases of blindness or low vision in countries where BD has a high prevalence. Several data have shown an improvement in visual prognosis in treated patients. The oldest studies, before the 1980s, showed an extremely poor visual prognosis. Mamo demonstrated in 1970 that in 39 BD patients, the average length of time for blindness in the right or the left eye was approximately 3.6 years [9]. A lower rate of visual loss was described in patients managed after 1990, interpreted as a reflection of the availability of cyclosporin A. The risk of vision impairment at 5 and 10 years in male and female patients was 21% versus 10% and 30% versus 17%, respectively [10]. The same team reviewed the records of patients managed in 1990–1994 and in 2000–2004. Visual acuity at three years was 20/200 or worse in 43/156 (27.6%) eyes in the first group and 26/201 (12.9%) eyes in the second group (*p* < 0.001); this trend was explained by an earlier aggressive therapy notably using conventional Disease-Modifying Antirheumatic Drugs (cDMARDS) and biologics [11]. However, based on most recent series, the blindness rate remained between 11 and 25% [12].

## 5. Diagnosis of Uveitis Associated with Behçet’s Disease

The diagnosis of BD remains a clinical diagnosis of exclusion in the absence of specific biological or histological markers. In incomplete forms, particularly in the absence of cutaneous and mucosal lesions or in patients with inaugural uveitis, the diagnosis is difficult, whereas the visual prognosis depends on the rapid initiation of appropriate treatment. In low-prevalence areas, the diagnosis may not be established, especially since uveitis with hypopyon is frequent in uveitis associated with HLA-B27 and other multiple etiologies may cause retinal vasculitis. It is therefore essential to be able to diagnose BD as early as possible by recognizing the associated ophthalmologic features and applying specific and relevant diagnostic criteria both for ocular involvement and systemic disease.

### 5.1. Diagnosis of Systemic Behçet’s Disease

BD diagnosis is based on clinical classification criteria. The key mucosal features in BD are oral and genital ulcers, recurrent and disabling [7,13]. Other skin lesions include pseudofolliculitis or erythema nodosum. Joint involvement is mostly non-erosive monoarthritis. However, BD is often not diagnosed until several years after the appearance of the onset sign [14]. Several classification criteria for BD diagnosis exist. The International Study Group (ISG) criteria, established in 1990, required the presence of oral ulcers and at least two other items, including genital ulcer, dermatological lesion, i.e., pseudofolliculitis and/or erythema nodosum, ocular involvement and/or pathergy phenomenon [15]. Its sensitivity has been estimated at 85%, linked to the fact that criteria could fail to recognize atypical and/or early BD clinical presentation, especially since the criterion of oral ulcers is mandatory [16]. To improve its sensitivity, these criteria were revised in 2013, in the International Criteria for the Classification of Behçet’s Disease (ICBD), which assigns a score of 2 points each for ocular lesions, oral ulcers and genital ulcers, and 1 point each for skin lesions, central nervous system involvement and vascular manifestations. The pathergy test, when used, was assigned 1 point. A patient scoring ≥ 4 points is classified as having BD with an estimated sensitivity of 94.8% [17]; the aim is to obtain a definite and earlier diagnosis in order to avoid severe complications by referring patients to expert centers to begin appropriate treatment. In countries where BD is rare, like France, diagnosis seems more probable in patients from geographical areas where BD is highly prevalent; likewise, BD family history increases the likelihood of diagnosis [7].

### 5.2. Diagnosis of Uveitis Associated with Behçet’s Disease

#### 5.2.1. Clinical Characteristics and Investigations

Uveitis is the most frequent form of ocular involvement and is described in 28 to 70% of patients according to the literature [1,18]. Both the anterior and posterior segments of the eye may be affected. Nonetheless, other uncommon presentations of ocular involvement are described, such as conjunctival ulcers, episcleritis, scleritis, keratitis, isolated optic neuritis, papilledema, orbital inflammation and extraocular muscle palsies. The age at onset is between 20 and 30 years, rarely at 50 years or older. Panuveitis is the most frequent presentation and is more commonly found in men. Ocular involvement is mostly bilateral, i.e., around 80%, and can be the initial presentation. Bilateralization can occur and may be rapid, on average 2 years. A large retrospective Turkish study of 880 patients with BD uveitis has shown that male patients have a younger age at onset and more severe disease [10].

BD uveitis has several distinctive clinical features that distinguish it from other uveitic entities and from other systemic autoimmune diseases. Ocular uveitis is characterized by recurrent flares of intraocular inflammation. Isolated anterior uveitis is rare and affects less than 15% of patients. It is clinically manifested by sudden acute onset, visual acuity decrease, ocular redness, periorbital pain, photophobia and tearing. BD uveitis is always non-granulomatous, associated with anterior chamber flare and cells. It may be complicated by posterior synechiae. Hypopyon reflects the severity of uveitis. The incidence of hypopyon in other large series of BD uveitis ranges from 5.4 to 32.4% [19]. Recurrence of anterior uveitis may be complicated by glaucoma. Ocular hypertonia is the result of angle closure by anterior synechiae or pupillary closure, inflammation or local or systemic corticosteroid administration [12].

**Figure 1:**
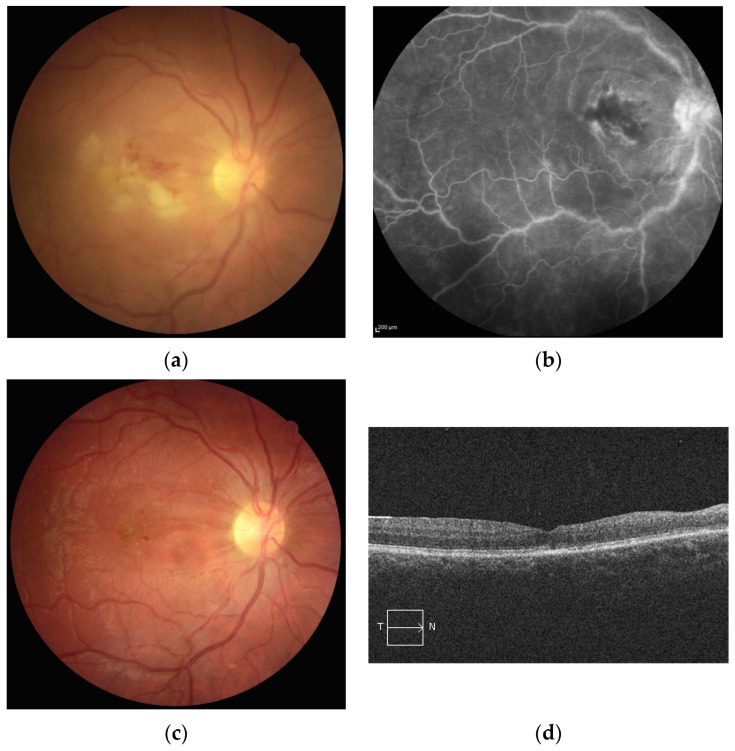
(**a**) Retinal photography of the right eye of a patient with Behçet’s disease showing a white infiltrate in the inter-papillomacular area associated with retinal edema and hemorrhage. (**b**) Fluorescein angiography showing vascular staining in the involved area. (**c**) Two months later, retinal photography showing retinal and optic atrophy with resolution of the infiltrate. (**d**) Two months later, spectral domain OCT scan showing atrophy of the inner retinal layers.

Posterior ocular involvement is the most frequent and the most severe form of uveitis, as it affects the visual prognosis. It manifests itself by an isolated decrease in visual acuity or may be asymptomatic. It can present white-yellowish, hemorrhagic retinitis areas of variable number and distribution. Their presence may be associated with a severe loss of vision when the macular area is involved (Figure 1).

Vitreous haze and cells may limit access to the fundus. Vasculitis in BD is frequent and most of the time venous but can be arterial or both. BD vasculitis is classically occlusive in nature [19]. These peripheral ischemic areas may be complicated by preretinal or papillary neovascularization, which may cause retinal hemorrhage, vitreous hemorrhage or neovascular glaucoma. Macular edema may occur and affect the visual prognosis. In the case of significant bilateral papilledema, cerebral imaging should be performed to detect cerebral thrombophlebitis or inflammatory neuropathy. Capillaropathies are seen on fluorescein retinal angiography [12]. Complications caused by recurrent posterior inflammatory flares include retinal atrophy, vascular sclerosis, optic atrophy, neovascular glaucoma and retinal detachment [3].

Identification of ocular posterior segment involvement is essential for the diagnosis, to define severity and prognosis and to monitor response to treatment. Sequential retinography of transient retinal lesions such as vasculitis or retinal necrosis areas can guide the diagnosis. Moreover, localized retinal nerve fiber layer defects not associated with a retinochoroidal scar, in the absence of glaucoma, could guide diagnosis of BD uveitis. They are linked to past foci of retinitis, which are transient and resolve without scar formation, and so could be missed [20].

**Figure 2:**
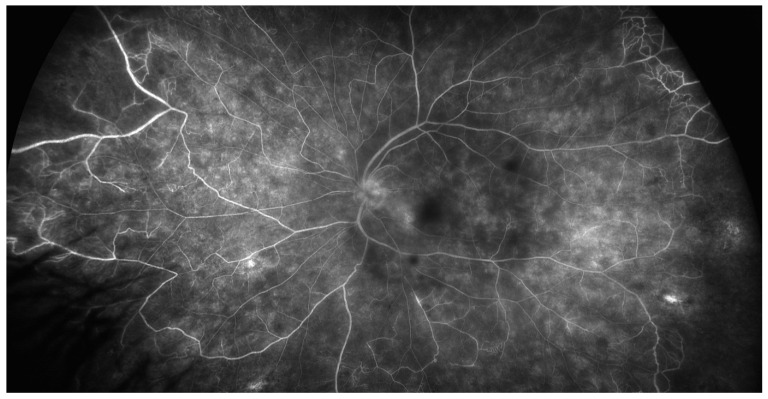
Late phase of ultra-wide field fluorescein angiography showing papillary hyperfluorescence, extensive retinal capillaropathy and peripheral occlusive vasculitis in Behçet’s disease uveitis.

**Figure 3:**
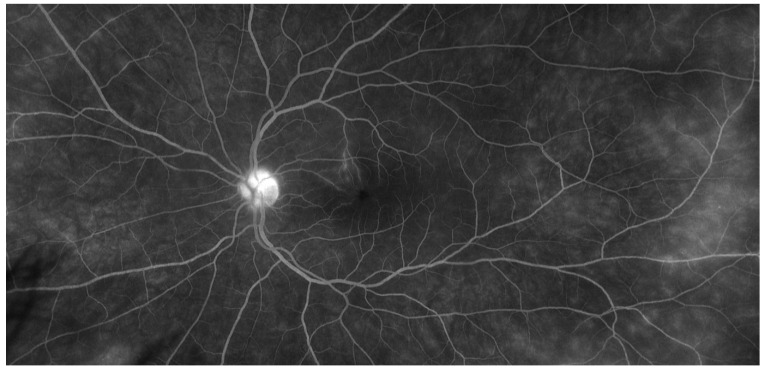
Late phase of ultra-wide field fluorescein angiography showing peripheral capillaropathy and vasculitis in the macular area responsible for visual acuity loss during Behçet’s disease uveitis.

Fluorescein angiography (FA) is the gold standard imaging modality for retinal vasculature. FA is a mandatory tool for the assessment of inflammatory fundus conditions due to posterior uveitis; the leakage on FA identifies retinal vasculitis and is a crucial marker for BD uveitis activity [21]. Specific signs of inflammatory activity include increased retinal vein tortuosity, vessel wall staining and leakage from retinal vessels and from the optic disc (Figure 2 and Figure 3). Fern-like capillary leakage is the most characteristic FA finding of BD uveitis and may be present even when uveitis seems inactive.

Optical Coherence Tomography (OCT) is based on an optical phenomenon, combining the analysis of wavelengths of reference light and light reflected by the structures of the eye. OCT produces axial section images of the fundus. OCT is complementary to FA, in particular to diagnose and to monitor macular complications such as macular edema, retinal cysts, retinal serous detachment, epiretinal membranes, vitreomacular traction, foveal atrophy and macular holes [22].

#### 5.2.2. Strategy for Definite and Earlier Diagnosis of Behçet’s Disease Uveitis

As described above, BD uveitis diagnosis is usually established in the presence of a coherent ocular clinical presentation and extraocular manifestations according to classification criteria. The different symptoms of BD can develop over several years [23]. Furthermore, uveitis could be the first manifestation of the disease, reported in 6–20% of patients [10,24,25], when extraocular manifestations of the disease may not yet have appeared. Recently, Tugal-Tutkun et al. proposed an algorithm to allow BD uveitis to be diagnosed solely on ophthalmological criteria. In this study, the most relevant signs for the BD uveitis diagnosis in patients with vitritis were: presence of foci of retinitis, occlusive retinal vasculitis, diffuse retinal capillary leakage on FA and absence of granulomatous anterior uveitis or choroiditis. However, these findings need to be confirmed in larger patient cohorts. Even though relapsing-remitting course showed a high clinical value, this criterion was not relevant in this retrospective evaluation because patients were treated before spontaneous resolution [26].

**Figure 4:**
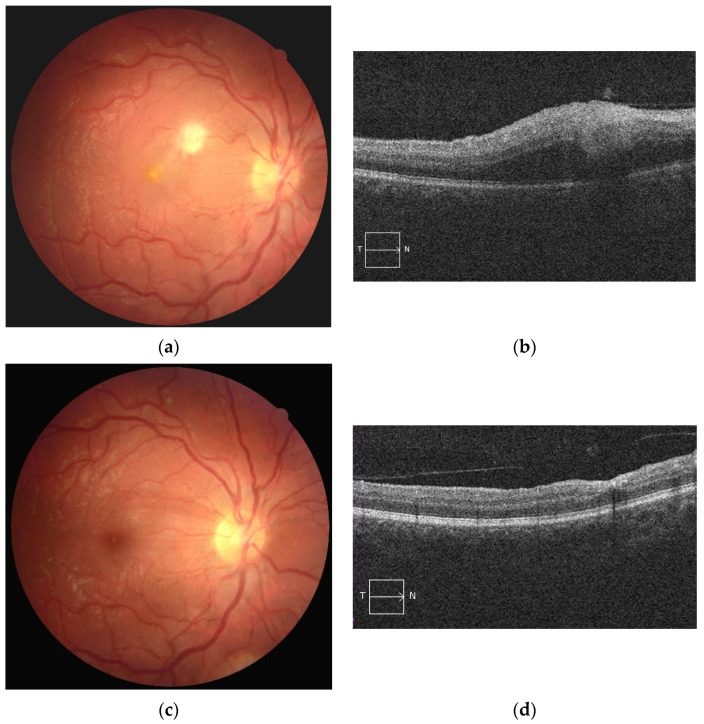
(**a**) Retinal photography of the right eye showing area of whitish retinal necrosis related to Behçet’s disease and (**b**) its appearance on OCT as focal inner nuclear layer thickening; (**c**) its resolution 2 months later and (**d**) its appearance on OCT as focal inner nuclear layer collapse.

FA remains the gold standard to diagnose and monitor BD uveitis. However, it is a time-consuming procedure that requires the injection of a fluorescent dye, which may be associated with severe allergic reaction. As a result, FA in clinical practice cannot be performed as often as necessary to best monitor the activity of patients with BD uveitis and therapeutic adaptation. In contrast, OCT is a non-invasive tool used to visualize the fundus. Furthermore, OCT can provide useful markers of previous posterior ocular flares such as outer plexiform layer elevations associated with focal inner nuclear layer collapse (Figure 4) [27]; this could help with retrospective diagnosis in case of clinical suspicion.

A more recent technology, Enhanced Depth Imaging OCT (EDI-OCT) provides detailed and measurable images from the choroid. A recent study demonstrated that subfoveal choroidal thickness might reflect macular vasculitis or inflammation; its measurement could be a noninvasive tool to investigate macular inflammation activity in BD uveitis [28]. However, it should be noted that a study with contradictory results, showing no increase in thickening of choroid during active BD uveitis, was also published [29].

A major limitation of FA is its inability to image the entire retinal capillary system. Optical Coherence Tomography Angiography (OCTA) is an innovative and recent technique in ophthalmology. OCTA is a fast, noninvasive diagnostic imaging technique, detecting movement in blood vessels, without dye injection and allows depth-resolved visualization of the retinal and choroidal vasculature. Several studies have recently investigated its role in describing microvascular changes in BD uveitis. OCTA has been found to allow better visualization of microvascular macular area changes such as capillary dropout, increased foveal avascular zone, telangiectasias, shunts and areas of neovascularization in comparison with FA in eyes with active BD uveitis. The deep capillary plexus seems to be more affected than the superficial capillary plexus [30,31,32]. However, FA remains essential to detect retinal vascular and capillary leakage at the macular area and at the peripheral retina and to affirm uveitis activity [33]; although OCTA can detect areas of retinal ischemia, it is not able to identify retinal vasculitis.

Furthermore, several recent publications analyzed with interest changes in retinal microvascularization in BD patients without uveitis, before the emergence of evident clinical findings. Parafoveal microvasculature seems to be involved in BD patients with uveitis and in BD patients without uveitis [34,35,36,37]; likewise, peripapillary microvascular changes could be detected by OCTA in BD patients without clinical ocular involvement [38]. FA was performed to ensure absence of any vascular leakage or subclinical vasculitis [34]. In one study published in 2019, no difference was observed in any measurement between BD patients without uveitis and healthy controls (51 eyes from BD patients without uveitis vs. 53 eyes from healthy individuals) [39]. Large-scale studies are needed to clarify the role of OCTA. However, this non-invasive and rapid technique seems to provide a benefit for BD patients in diagnosis, follow-up and prognosis of associated uveitis and could even provide an additional argument for patients with suspected BD. It can also improve the assessment of a known disease without ocular damage and can consequently modify the treatment decision.

Conventional color retinography and FA are limited in their field of view. Most fundus cameras can capture a maximum of 60° of the entire fundus at a time. While a mosaic can be performed to enlarge field of view, the entire fundus cannot be imaged simultaneously. Ultra-wide-field imaging, providing a 200° angle of photographic, autofluorographic and angiographic ocular fundus views, has recently been introduced in ophthalmology. In a prospective observational study of 23 patients with non-infectious retinal vasculitis, the authors showed that disease activity and uveitis management were changed when ultra-wide-field angiography was added to standard imaging (60°) [40]. In the future, it is likely to become an essential tool for the diagnosis, treatment and follow-up of retinal vasculitis, in particular when associated with BD. However, additional studies are needed to clarify how this improved identification of otherwise unrecognized peripheral retinal changes will impact treatment decisions, patient prognosis and outcomes [41,42].

The laser flare meter can be used to monitor the degree of inflammation, as its values would correlate with the amount of vascular leakage visible on FA [43].

## 6. Treatment Modalities and Perspectives

### 6.1. Treatment Aims

BD prognosis is dominated by ocular, neurological and vascular damage, with a poor functional and/or vital prognosis. Ocular involvement is severe and frequent, rapidly involving the visual prognosis. The incidence of ophthalmological impairment in BD patients is close to 70% [3]. It is characterized by acute flare-ups that may regress spontaneously. During periods of remission, the eyes are calm or mildly inflammatory. The objectives of therapeutic management of BD uveitis are to rapidly and effectively control inflammation in order to preserve visual function and limit irreversible structural damage, but also to treat the chronic subclinical inflammation, to prevent relapses and ocular complications, to limit ophthalmological and general side effects of iatrogenic causes and to control the associated systemic manifestations [44,45]. A multidisciplinary team and approach are essential. Furthermore, care and follow-up of ocular involvement should be handled by an ophthalmologist familiar with chronic ocular inflammatory diseases.

### 6.2. Conventional Immunosuppressants

Azathioprine and cyclosporin A are the only two immunosuppressants evaluated in randomized controlled trials (RCT). Azathioprine (2.5 mg/kg/day), in a large placebo-controlled trial, led to significantly reduced hypopion uveitis relapses and new eye disease after 24 months. In the azathioprine group, no serious adverse events were reported, whereas in the placebo group, one patient died of pulmonary artery aneurysm [46]. Cyclosporin A was evaluated in three RCT [47,48,49]. The response rates reported were between 80 and 91%, but side effects were frequent [47,48,49,50,51]. Cyclosporin A was more effective than cyclophosphamide [49]. However, its side effects, in particular nephrotoxicity, may currently limit its use in an uveitis context [52]. A 15-year longitudinal study using methotrexate (7.5–15 mg/week) showed visual acuity improvement and decrease in 46.5% and 37.2% of patients with BD uveitis, respectively [53]. However, methotrexate, mycophenolate mofetil and tacrolimus are not usually used in BD uveitis management. Alkylating agents are not recommended anymore due to their safety profile, in particular, malignancies and infertility. Biologics appear to be more effective and safer [54].

### 6.3. Interferons

Interferons are cytokines that can be synthesized by most cells and have antiviral, antiproliferative and immunomodulatory properties. Their efficacy and tolerance were analyzed in BD patients [55,56,57]. Several studies emphasized the efficacy and tolerance of interferon α2a (IFN-α2a) in adults and in children with BD uveitis [58,59,60,61,62,63,64]. First treatment modalities, i.e., doses ranging from 3 to 9 million UI daily versus thrice a week regimen, duration administration and corticosteroid tapering vary widely among studies. Subcutaneous IFN-α2a (3 million UI thrice a week) is effective and safe for the long-term management of refractory BD uveitis. It has a potent corticosteroid-sparing effect [65]. A partial or complete response is estimated in around 90% of treated BD uveitis patients [54]. Moreover, it would also allow in some cases long-term remission even after discontinuation [66,67,68]. However, IFN-α2a production was recently stopped and it is no longer commercially available. Pegylated interferon-alpha-2a (PEG-IFN-α2a), administered once a week, remains available. In an RCT, adding PEG-IFN-α2a to the drug regimen of BD patients with or without ocular involvement did not significantly reduce the cortico-dependence threshold at 1 year. However, in patients on corticosteroids at baseline, post hoc analysis demonstrated that adding PEG-IFN-α2a reduced the corticosteroid dose required for significant improvement in quality of life [69]. Small case series reported IFN-α2b or IFN-α2a efficacy in BD uveitis [70,71], even though IFN-α2a was described to be more effective than IFN-α2b [72]. Further studies are needed on the pegylated form efficacy on active disease and maintenance therapy of BD uveitis. The main limitation of interferon treatment is tolerance, with the occurrence of a frequent flu-like syndrome and severe psychological disorders that can lead to suicide attempts [66]. However, compared with anti-TNFα agents, it does not promote severe infection, especially tuberculosis.

### 6.4. Anti-TNFα Agents

Biologics have dramatically changed BD uveitis management, although their use is based on uncontrolled studies with few randomized controlled trials [73]. In a recent retrospective study, ocular inflammation was controlled earlier with anti-TNFα agents compared to cDMARD in non-anterior non-infectious uveitis, and better corticosteroid sparing was achieved [74]. Currently, five anti-TNFα agents are available; infliximab and adalimumab are the two mainly used in BD uveitis.

Infliximab is a murine–human chimeric monoclonal antibody (mAb) against soluble and transmembrane forms of TNFα. In BD, the usual dose is 5 mg/kg, intravenously, which may be increased to 10 mg/kg. Infusions are repeated after 2 and 6 weeks and then every 4 to 6 weeks. Infliximab was first reported for ocular inflammation in BD in 2001 [75]. Then, the published evidence consisted mainly in reports of the open use of infliximab. A retrospective comparison of infliximab vs. cyclosporin A during the first 6-month treatment period in BD showed infliximab to be more effective in reducing acute episodes of BD uveitis, although no significant improvement was observed in visual acuity [76]. After infliximab, a rapid improvement of visual acuity and decrease of ocular inflammation starting at 24 h was almost always reported among 158 patients [77]. In 89% of these patients, significant reduction of uveitis relapses was observed. Despite the lack of RCTs, infliximab was approved in Japan for the treatment of “Behçet’s disease complicated with refractory uveoretinitis, which does not respond to conventional therapies” (Osaka, Japan, 26 January 2007, JCN Newswire). Moreover, a prospective comparative study comparing acute panuveitis relapses in BD management showed that infliximab (5 mg/kg), at the onset of uveitis, is significantly faster in controlling ocular inflammation than intravitreal triamcinolone (4 mg) or high-dose methylprednisolone (3 day, 1 g/d) [78]. Control of acute ocular inflammation in BD is mandatory to avoid permanent loss of vision; therefore, an intravenous infliximab infusion should always be considered for BD panuveitis relapses. No studies comparing infliximab and IFN-α2a have been published; however, a recent meta-analysis showed similar remission rates, with a sustained remission rate higher in the IFN-α2a group (71%) compared to infliximab (43%). Infliximab has a more rapid onset of action; rates for improving visual acuity were 46% for IFN-α2a and 76% for infliximab. Withdrawal rates due to side effects were similar, i.e., 5.5% (INF-α2a group) vs. 5% (infliximab group) [79].

Adalimumab is a fully human mAb binding TNFα. The benefit of adalimumab is subcutaneous administration at a dose of 40 mg every 2 weeks in adults after a loading dose of 80 mg in uveitis. Adalimumab was approved in 2016 for use in the management of non-infectious intermediate, posterior and panuveitis. Adalimumab efficacy and safety in BD uveitis have been progressively reported. Adalimumab was first successfully used in case series [80,81,82], and then in several retrospective studies [83,84,85]. The two RCTs vs. placebo VISUAL I and VISUAL II evaluated efficacy and safety of adalimumab in active and inactive non-infectious uveitis of any etiology, respectively [86,87]; there were too few BD uveitis patients to be analyzed specifically.

A recent meta-analysis was conducted to assess the effectiveness and safety of anti-TNFα agents in the management of BD uveitis among 18 clinical trials, i.e., 3 prospective and 15 retrospective studies, from January 2010 to December 2019, with a minimum follow-up of 6 months and at least 10 patients with BD uveitis. The overall uveitis remission rate was 68% (95% CI 0.59 to 0.79), visual acuity improvement rate was 60% (95% CI 0.47 to 0.77) and central macular thickness decrease was 112.70 μm (95% CI 72.8 to 153.0) with a significant corticosteroid-sparing effect. In this review, 2.62% of patients had serious side effects [88]. Furthermore, Vallet et al. retrospectively evaluated the adalimumab and infliximab efficacy in 160 refractory uveitis, including 36% of BD uveitis. These two anti-TNFα agents appeared to be similar in terms of efficacy and BD uveitis was linked to a 3-fold increase in the complete response rate [89].

In case of failure of a first anti-TNFα agent, a switch to another can be useful. In 124 BD patients described by Vallet et al., 31 patients received a second line of anti-TNFα agent due to lack of efficacy, side effects or patients’ decision. Concerning ocular manifestations, complete and partial responses were reported in 12 (67%) and 5 (28%) patients, respectively [90]. An observational multicenter study was recently conducted, comparing the efficacy of infliximab and adalimumab as a first-line treatment in refractory BD uveitis. In both groups (103 infliximab patients and 74 adalimumab patients), after 1 year, all ocular parameters improved, with a significant difference in best-corrected visual acuity, anterior chamber inflammation and vitritis in the adalimumab group, compared to the infliximab group. However, a faster improvement of anterior chamber inflammation and vitritis was described with infliximab, even though the adalimumab group did not receive a loading dose. A higher retention rate was observed in the adalimumab group (95.24% vs. 84.95%; *p* = 0.042); infliximab and adalimumab were stopped due to lack of efficacy in 17.5% and in 14.9%, respectively. Interestingly, no significant difference was reported between the two treatments for improvement of vasculitis and macular edema [91].

The cumulative retention rate of adalimumab in 54 BD uveitis patients was evaluated at 76.9% at 12 months and at 63.5% at 48 months of follow-up. It was not modified by the use of concurrent DMARDs or by different lines of biologic agents. Moreover, retention rates were not lower in cases of known negative prognostic factors for BD ocular involvement, such as male gender, early age at disease onset and the duration of uveitis [92]. Similarly, the cumulative retention rates of infliximab in 40 BD uveitis patients at 12-, 24-, 60- and 120-month follow-up were 89.03%, 86.16%, 75.66% and 47.11%, respectively, not modified by the use of concomitant DMARDs or by negative known prognostic factors. A significantly lower retention rate was observed when infliximab was administered following other biologics. At 10-year follow-up, discontinuation was due to: secondary failure (six patients), primary failure (two patients), side effects (four patients), prolonged disease remission (two patients) and switch to subcutaneous treatment (one patient) [93]. Recently, Horiguchi et al. showed that infliximab monotherapy was effective and not inferior to combination therapies, i.e, colchicine or corticosteroids for refractory BD uveitis over a 10-year follow-up [94].

In case of inefficacy of infliximab, an increased dose or an increased frequency of administration was described in BD uveitis management [91]. Similarly, for adalimumab, an increased frequency of administration, i.e., weekly, was recently described in uveitis, with encouraging results but not specifically in this indication [95,96].

Etanercept is a fusion protein, which is a soluble receptor binding soluble TNFα and preventing it from binding to target cells. Etanercept has lower efficacy for uveitis treatment than anti-TNFα antibodies [97].

Golimumab is a fully human anti-TNFα mAb. Several case reports and series reported successful control of severe uveitis, especially in juvenile idiopathic arthritis and BD [98,99,100,101,102].

Certolizumab pegol is a pegylated recombinant humanized mAb against TNFα. Pegylation leads to its delayed elimination. Data on use of certolizumab-pegol for BD uveitis are currently limited.

Anti-TNFα agents are associated with an increased risk of tuberculosis [103]. Screening for latent tuberculosis is recommended for all patients before starting therapy. Severe opportunistic infections, in particular those with intracellular microorganisms, may occur. Demyelinating events, including exacerbations of preexisting multiple sclerosis, were reported [104,105,106,107,108]. There is no evidence for an increased risk of solid tumor or lymphoproliferative disease with anti-TNFα agents [109,110], except non-melanoma skin cancer [111]. All anti-TNFα agents can induce antinuclear antibodies; however, the development of anti-TNFα-induced lupus is more rarely reported. Local complications at the drug administration site have been frequently observed. Anti-TNFα agents may induce neutralizing antibodies, resulting in loss of efficacy and the appearance of infusion reactions [112]. New onset and worsening of congestive heart failure have been described [109]. Sarcoidosis is a rare and paradoxical side effect during anti-TNFα treatment, as well as occurrence of psoriasis [97].

### 6.5. Peri or Intraocular Treatment

Intravitreous or periocular injections of corticosteroids as adjuvant treatment in addition to systemic treatment could be proposed in the event of unilateral flare. Intra-ocular pressure elevation and cataract development are the main side effects in addition to the absence of systemic disease control. This option can be used as a bridging therapy awaiting escalation in therapy or in rare cases of absolute contraindication to some systemic therapies [113].

A first study of 15 patients with BD uveitis treated with intravitreal infliximab injections (1.5 mg intravitreal infliximab) showed a significant improvement in visual acuity, with a significant decrease in retinal vasculitis, retinitis and macular thickness [114]. Similarly, a second study showed that intravitreal infliximab appeared to be safe and effective in treating uveitis in 20 BD patients [115]. However, contradictory results regarding its safety and efficacy have also been reported in a more recent study in 16 patients. Four eyes developed severe immunological reaction and failure to control inflammation was described in the majority of eyes [116]. Intravitreal adalimumab was not successful in chronic refractory cystoid macular edema, i.e., reduction of central retinal thickness and improvement of visual acuity [117]; no ocular or systemic adverse events were observed. Then, intravitreal adalimumab was shown in a very small patient population to be effective in improving the best-corrected visual acuity, controlling inflammation, limiting uveitis flare and decreasing macular edema in non-infectious uveitis, including BD uveitis [118,119]. Otherwise, contradictory results concerning the safety of intravitreal adalimumab have been reported [117,120,121,122]. Further studies regarding the concentration and toxic effects of intravitreal anti-TNFα agents are required, although their efficacy is uncertain.

Intravitreal bevacizumab was shown to be well tolerated and an effective supplementary therapy for chronic uveitis cystoid macular edema, particularly in BD; however, the median period of efficacy was short [123].

### 6.6. Retinal Laser Photocoagulation

Development of optic disc neovascularization is a severe complication of BD uveitis. Although scatter laser photocoagulation may be necessary in eyes with extensive retinal ischemia, inflammatory mechanisms seem to be essential in its pathogenesis. Therefore, in these cases, treatment intensification is appropriate and laser photocoagulation should be avoided [44].

### 6.7. BD Uveitis Management Recommendations

Due to its severity, BD uveitis was the first uveitis for which anti-TNFα agents were recommended [124]. EULAR recommendations on BD treatment were recently updated [79]; likewise, French recommendations were recently proposed [7,125]. In case of posterior segment ocular involvement, glucocorticoids should never be used alone. Systemic immunosuppressive agents such as azathioprine, cyclosporin A, IFN-α, infliximab or adalimumab should be proposed, depending on the risk of infection such as tuberculosis with anti-TNFα agents, tolerability of IFN-α2a, physicians’ experience and reimbursement policies. Patients presenting with an initial or recurrent episode of acute sight-threatening uveitis should be treated with high-dose glucocorticoids and infliximab or interferon-alpha [79]. French recommendations stated that in case of sight-threatening involvement, patients must be treated with high doses of corticosteroids associated with anti-TNFα or IFN-α; intravenous infliximab (5 mg/kg at weeks 0, 2, 6 and then every 4 to 6 weeks) or subcutaneous adalimumab (80 mg dose initially, then 40 mg/15 days) can be used [7]. Intravitreal corticosteroid injection is a therapeutic option in patients with unilateral flare as an adjunct to systemic treatment [7,79].

In isolated anterior uveitis, treatment is based on topical corticosteroids. However, systemic immunosuppressants could be considered, such as azathioprine, if there are factors of higher risk of more severe disease, such as young age, early onset of the disease and male gender [7,79].

**Figure 5:**
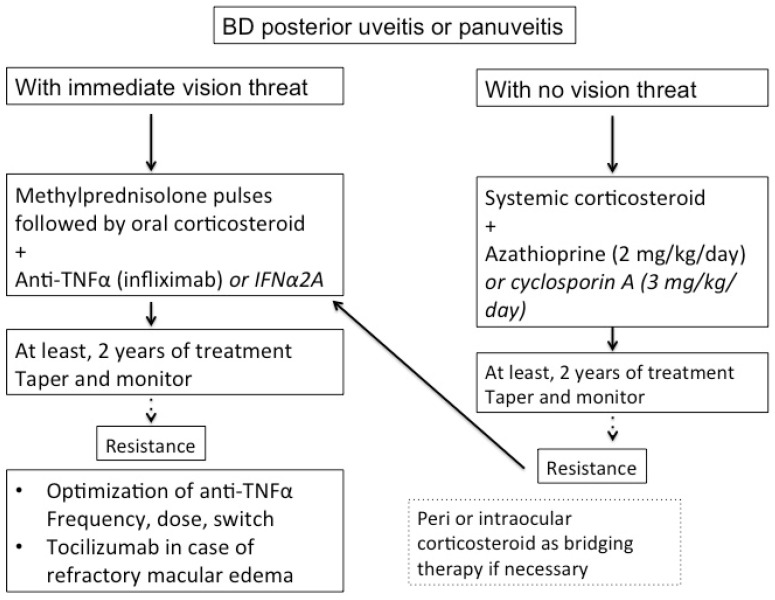
Proposed algorithm for management of severe Behçet’s disease (BD) uveitis.

During BD uveitis management, decrease or withdrawal of immunosuppressive or immunomodulating treatment should be considered only after a 2-year remission, and an expert examination is advised [7]. How to stop immunosuppressive drug in BD uveitis has not yet been defined. Martín-Varillas et al. proposed a strategy to progressively taper adalimumab in BD, by increasing the delay between each injection by one week, which allowed the control of ocular inflammation and resulted in fewer side effects [126]. Figure 5 summarizes the management of severe BD.

### 6.8. Biologics beyond Anti-TNFα Agents

#### 6.8.1. Anti-Interleukin-6 Agents

Tocilizumab (TCZ) is a humanized anti-interleukin-6 (IL-6) receptor mAb, inhibiting IL-6 pathway to prevent IL-6 from binding to its receptor. TCZ is authorized worldwide for various inflammatory diseases, such as rheumatoid arthritis, giant cell arteritis and Still’s disease. It is available intravenously monthly or subcutaneously weekly. IL-6 inhibitors may be effective in the management of posterior uveitis and macular edema in BD uveitis. In the prospective STOP-Uveitis study, intravenous TCZ was found to be safe and equally effective in both naïve and previously treated patients with non-anterior non-infectious uveitis, mostly idiopathic, i.e., one BD uveitis among 37 [127]. TCZ has also shown, in a retrospective study, its efficacy in 5 cases of BD uveitis refractory to IFN-α and anti-TNFα agents, when administered intravenously, i.e., 8 mg/kg [128], and in 11 cases of BD uveitis refractory to anti-TNFα agents [129]. In this last study, TCZ was only effective in extraocular manifestations among three patients. Furthermore, a recent review concluded that TCZ represented a promising therapy for refractory ocular-, neuro- and vasculo-BD, but was not recommended for mucocutaneous and articular involvement [130]. TCZ seems to be useful in refractory macular edema treatment associated with non-infectious uveitis. Vegas-Revenga et al. showed, in a multicenter retrospective study, significant macular edema reduction and visual acuity improvement in 25 patients, including 7 with BD uveitis [131].

An increased risk of infection is the most frequent serious side effect reported, especially gastrointestinal events. Anti-IL-6 agents have been associated with elevations in serum concentrations of transaminases, pancreatitis, gastrointestinal perforations in patients with preexisting risk factors and increased serum lipid concentration [132]. Interestingly, a study in rheumatoid arthritis patients showed a low immunogenicity of TCZ, whether or not there was an association with conventional immunosuppressants [133].

#### 6.8.2. Anti-Interleukin-1 Agents

Three anti-interleukin-1 (IL-1) agents have been studied in BD treatment: anakinra, an IL-1 receptor antagonist protein; canakinumab, a human anti-IL-1β mAb; and gevokizumab, a humanized anti-IL-1β mAb. Their place in the management of BD uveitis remains unclear, based on contradictory findings in the literature. In a recent randomized, double-masked, placebo-controlled trial in patients having recently experienced a BD uveitis exacerbation (40 subcutaneous gevokizumab vs. 43 placebo), gevokizumab did not significantly reduce the median time to relapse [134]. However, in a previous open-label study, a single gevokizumab infusion resulted in a rapid and sustained reduction in ocular inflammation in seven patients with refractory BD uveitis [135]. These encouraging results were then confirmed in a prospective, open-label, randomized phase 2 trial in 21 BD uveitis patients treated with gevokizumab every 4 weeks intravenously or subcutaneously [136].

Furthermore, anakinra showed control of ocular inflammation in three out of four BD uveitis refractory to anti-TNFα agents. However, patients experienced relapse over time [137]. In a retrospective multicenter study, anakinra and canakinumab were shown to be efficacious and safe in 73% out of 30 patients, including 16 with BD uveitis, even though patients could benefit from therapeutic adaptation or switch to the other anti-IL-1. Side effects were, in all cases, represented by local cutaneous reactions [138]. In an observational study, anakinra or canakinumab were studied in 19 BD uveitis and improved retinal vasculitis and decreased the rate of uveitis relapses, i.e., from 200/100 patients per year to 48.87/100 patients per year during 12 months; no significant effect was measured in macular thickness and visual acuity [139].

#### 6.8.3. Anti-Interleukin-17 Agents

In active BD uveitis, anti-interleukin-17A (IL-17A) has been found to be significantly upregulated in human peripheral blood mononuclear cells. Secukinumab, the only anti-IL-17 agent studied in uveitis management to date, is a fully human mAb [140]. It was studied subcutaneously against placebo in three RCTs. The SHIELD study analyzed 118 non-anterior BD uveitis, while the INSURE study analyzed 31 non-anterior non-BD uveitis and the ENDURE study analyzed 125 quiescent non-anterior non-BD uveitis. In the SHIELD study, the primary endpoint, i.e., the reduction in rate of uveitis recurrence did not meet statistical significance, as in the other two studies. However, the secondary efficacy data from SHIELD and INSURE could suggest a potential benefit of secukinumab in reducing the use of concurrent immunosuppressants [141].

Nevertheless, a prospective study suggested the efficacy of intravenous secukinumab in active chronic non-infectious uveitis which required systemic immunosuppression in 16 patients, including one BD uveitis [142]; similarly, in a subsequent prospective study, intravenous secukinumab was observed to be more effective and tolerated in patients with non-infectious uveitis who required corticosteroid-sparing immunosuppressive therapy, compared with subcutaneous secukinumab in 37 patients without BD uveitis [143].

In a retrospective multicenter study evaluating 15 BD patients, refractory to treatment with colchicine, DMARDs and at least one anti-TNFα agent, the efficacy and safety of secukinumab have been reported in the treatment of mucosal and articular manifestations. At the time of secukinumab introduction, one patient with active anterior uveitis did not experience ocular relapse throughout follow-up [144].

These conflicting results may indicate that the strategy of blocking Il-17 may still be useful in the management of uveitis, probably by adjusting the dose received and controlling plasma therapeutic levels according to the route of administration.

#### 6.8.4. Anti-Interleukin-12/23 Agents

Ustekinumab is a fully humanized mAb with high affinity for IL-12 and IL-23, which seems to play a crucial role in non-infectious uveitis [145]. Promising results for uveitis associated with inflammatory bowel disease and psoriatic arthritis were reported [146,147,148]. Efficacy data on BD uveitis is not yet known. STELABEC-2, a phase 2 open-label study evaluating active posterior and pan-uveitis in BD uveitis, has finished recruiting patients, but results are not yet published (clinicaltrials.gov). Moreover, ustekinumab seems to be effective in controlling colchicine-resistant oral ulcers associated with BD [149,150].

#### 6.8.5. Other Biologics

Rituximab, B cell targeted therapy, is a chimeric mAb against CD20. Davatchi et al., in 2010, successfully tested, in a single blind randomized control study, the efficacy of rituximab associated with methotrexate vs. a combination of pulse cyclophosphamide and azathioprine in improving BD ocular involvement; no significant difference was observed between groups [151].

Alemtuzumab is a humanized mAb anti-CD52, able to induce a rapid and long-term B and T cell depletion. In a first study, 18 patients received a single infusion of alemtuzumab, including five BD patients with ocular involvement; complete or partial remission was described for all these patients at 6 months of follow-up. All patients received an antifungal and antiviral prophylactic treatment [152]. In another retrospective long-term study, 32 patients with BD received 60 courses of alemtuzumab between 1994 and 2013. Twenty-one patients presented ocular involvement and all of them achieved remission. Thyroid dysfunction was a frequent side effect, i.e., seen in 25% of patients. Antiviral and antifungal prophylaxis was also systematically administered [153].

Abatacept, T cell targeted therapy, is a recombinant fusion protein which can block CD-80 and CD-86 on antigen-presenting cells, necessary for its activation. Short-term efficacy was described in a case report of refractory BD-associated scleritis [154].

Daclizumab is a humanized mAb binding the CD25 unit of IL-2 receptor, which was studied during a randomized, placebo-controlled trial in 17 BD patients. Daclizumab was not superior in preventing relapse and tapering immunosuppressive drugs compared with placebo [155]. In 2018, it was withdrawn from the market after reports of autoimmune encephalitis [156].

#### 6.8.6. Targeted Synthetic Disease-Modifying Antirheumatic Drugs

Targeted synthetic Disease-Modifying Antirheumatic Drugs (tsDMARDs) comprise several types of agents such as phosphodiesterase inhibitors and kinase inhibitors. Their small size gives them a high level of bioavailability and moreover tsDMARDs have a low rate of immunogenicity [157].

##### Anti-Janus Kinase

Tofacitinib is an anti-Janus Kinase (JAK) 1/3 inhibitor, approved for autoinflammatory diseases such as rheumatoid arthritis, ulcerative colitis and psoriatic arthritis. A first study of 13 patients with refractory BD suggested its safety and possible efficacy in vascular and articular involvement. No ocular involvement was described and patients with gastrointestinal-BD responded poorly [158]. In a case series including two patients, tofacitinib appeared to be a potential new treatment option for refractory, non-infectious idiopathic uveitis or scleritis [159]. Encouraging preliminary results have recently been reported in uveitis associated with juvenile idiopathic arthritis [160,161].

##### Apremilast

Apremilast is a phosphodiesterase 4 inhibitor modulating cytokines that are upregulated in BD. Its efficacy has been demonstrated in BD oral ulcers in phase 2 and 3 randomized placebo-controlled trials and it is now approved in this indication. Nevertheless, its possible role in the management of BD uveitis has not been studied yet [162,163,164].

## 7. Conclusions

Despite diagnostic and therapeutic innovations, BD uveitis remains a severe condition. Biologics have transformed the management of intraocular inflammation, but in the absence of a reliable biological marker and possible subclinical ophthalmological involvement, the early diagnosis of BD remains a major challenge. Improvements in ocular multimodal imaging will probably allow a better evaluation of patients. However, there are still cases of BD uveitis that are refractory to the recommended treatment and studies comparing the different existing biologics will help to improve management. The therapeutic armamentarium is expanding, with probably useful alternatives to anti-TNFα or interferons. However, some questions remain unanswered, such as treatment duration and long-term strategies.
